# Furuncular Myiasis on Glans Penis

**DOI:** 10.4269/ajtmh.13-0688

**Published:** 2014-08-06

**Authors:** Marcelo Rosandiski Lyra, Bruno Cruz Fonseca, Nathasha Sbragio Ganem

**Affiliations:** Instituto de Pesquisa Clínica Evandro Chagas (IPEC), Fundação Oswaldo Cruz (Fiocruz), Rio de Janeiro, Brazil; Department of Infectious and Parasitary Diseases, Hospital Central do Exército (HCE), Rio de Janeiro, Brazil

**Figure 1.**
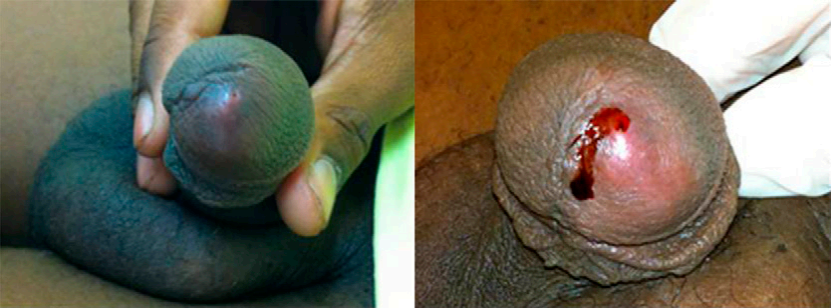
Nodule with central pore on the glans penis with serosanguinous discharge.

**Figure 2.**
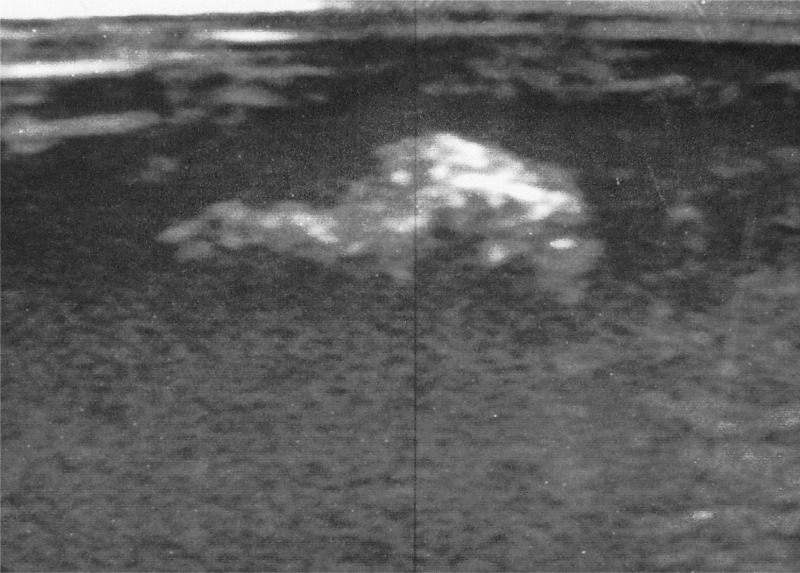
Ultrasound showed fusiform echogenic image.

**Figure 3.**
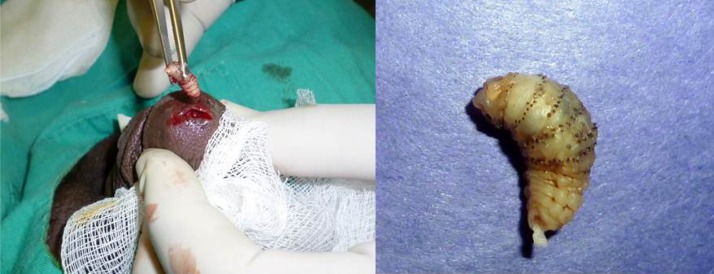
Surgical removal of *D. hominis* larva.

A 20-year-old military soldier from Rio de Janeiro, Brazil presented a nodule with a central pore on the glans penis with serosanguineous discharge, bilateral inguinal lymphadenopathy, and severe local pain (Figure 1). He had returned from a military mission in a rural area with poor hygiene conditions 2 weeks earlier. Ultrasound assessment with color flow Doppler showed a fusiform irregular echogenic image measuring 11 × 3 × 6 mm without urethra communication, confirming clinical suspicion of furuncular myiasis (Figure 2). He received ivermectin (200 μg/kg), and a *Dermatobia hominis* larva was surgically extracted (Figure 3).

Myiasis is an infestation of vertebrate hosts by larvae of flies that feed on living tissue, body fluids, or ingested foods. Furuncular myiasis is transmitted to vertebrate animals by a hematophagous insect, on whose abdomen a female botfly has deposited her eggs. When the blood-feeding vector encounters a warm-blooded animal, temperature change leads botfly eggs to hatch.^1^ Larvae enter the vertebrate host either through a hair follicle or the bite site or by directly burrowing in the skin, forming a nodular lesion. Penile myiasis is rare and can be confused with an STD, furunculosis, or glans abscess.^2^
